# Effects of CYP3A4*22 and CYP3A5 on clinical outcome in patients treated with ticagrelor for ST-segment elevation myocardial infarction: POPular Genetics sub-study

**DOI:** 10.3389/fphar.2022.1032995

**Published:** 2022-12-05

**Authors:** Jaouad Azzahhafi, Thomas O. Bergmeijer, Wout W. A. van den Broek, Dean R. P. P. Chan Pin Yin, Senna Rayhi, Joyce Peper, Willem L. Bor, Daniel M. F. Claassens, Ron H. N. van Schaik, Jurriën M. ten Berg

**Affiliations:** ^1^ Department of Cardiology, St. Antonius Hospital, Nieuwegein, Netherlands; ^2^ Department of Cardiology, Isala, Zwolle, Netherlands; ^3^ Department of Clinical Chemistry, Erasmus Medical Centre, Rotterdam, Netherlands; ^4^ Cardiovascular Research Institute Maastricht (CARIM), University Medical Center Maastricht, Maastricht, Netherlands

**Keywords:** acute coronary syndrome, ticagrelor, genetic testing, myocardial infarction, percutaneous coronary intervention, pharmacogenetics

## Abstract

**Aims:** To determine the clinical efficacy, adverse events and side-effect dyspnea of CYP3A4*22 and CYP3A5 expressor status in ticagrelor treated patients.

**Methods and results:** Ticagrelor treated patients from the POPular Genetics randomized controlled trial were genotyped for CYP3A4*22 and CYP3A5*3 alleles. Patients were divided based on their genotype. In total 1,281 patients with ST-segment elevation myocardial infarction (STEMI) were included. CYP3A4*22 carriers (*n* = 152) versus CYP3A4*22 non-carrier status (*n* = 1,129) were not found to have a significant correlation with the primary thrombotic endpoint: cardiovascular death, myocardial infarction, definite stent thrombosis and stroke [1.3% vs. 2.5%, adjusted hazard ratio 1.81 (0.43–7.62) *p = 0.42*], or the primary bleeding endpoint: PLATO major and minor bleeding [13.2% vs. 11.3%, adjusted hazard ratio 0.93 (0.58–1.50) *p = 0.77*]. Among the CYP3A4*1/*1 patients, CYP3A5 expressors (*n* = 196) *versus* non-expressors (*n* = 926) did not show a significant difference for the primary thrombotic [2.6% vs. 2.5%, adjusted hazard ratio 1.03 (0.39–2.71) *p = 0.95*], or the primary bleeding endpoint [12.8% vs. 10.9%, adjusted hazard ratio 1.13 (0.73–1.76) *p = 0.58*]. With respect to dyspnea, no significant difference was observed between CYP3A4*22 carriers versus CYP3A4*22 non-carriers [44.0% vs. 45.0%, odds ratio 1.04 (0.45–2.42) *p = 0.93*], or in the CYP3A4*1/*1 group, CYP3A5 expressors *versus* CYP3A5 non-expressors [35.3% vs. 47.8%, odds ratio 0.60 (0.27–1.30) *p = 0.20*].

**Conclusion:** In STEMI patients treated with ticagrelor, neither the CYP3A4*22 carriers, nor the CYP3A5 expressor status had a statistical significant effect on thrombotic and bleeding event rates nor on dyspnea.

**Clinical Trial Registration:** ClinicalTrials.gov, identifier NCT01761786.

## Introduction

Patients with acute coronary syndrome (ACS) are treated with dual antiplatelet therapy (DAPT), consisting of aspirin and a P2Y_12_ inhibitor, according to the current guidelines. It is estimated that, in 2015, the number of patients requiring DAPT has increased to approximately 1.4–2.2 million patients per year worldwide. Current ACS guidelines recommend the use of the stronger antiplatelet drugs ticagrelor or prasugrel over clopidogrel in combination with aspirin. The use of clopidogrel in patients with ACS is currently limited to the situation when ticagrelor or prasugrel are not available, cannot be tolerated, or are contraindicated (Ibanez et al., 2017; Collet et al., 2020).

Ticagrelor is a direct acting oral, reversible antiplatelet agent with a plasma half-life of approximately 7–12 h. Unlike clopidogrel, which has to be metabolized into its active variant by hepatic CYP450 enzymes in order to gain its therapeutic effect, ticagrelor does not require metabolic activation. However, ticagrelor is extensively metabolized and eliminated by primarily CYP3A4, and, to a lesser extent, by CYP3A5 (as shown in *vitro* studies) into the metabolites C124910XX and C133913XX (Liu et al., 2017a). Therefore, it is not recommended to combine ticagrelor with strong CYP3A4 inhibitors or inducers. For example, concomitant use of ketoconazole increases the C_max_ of ticagrelor 2.4 times and the Area Under the Curve (AUC) 7.3 times (Teng and Butler, 2013). The C124910XX compound exhibits almost the same potency in antiplatelet effect as the parent drug and is present at approximately 30%–40% of the levels of ticagrelor. C124910XX is further metabolized by UDP-glucuronosyltransferase, or *via* hydroxylation to a minor hydroxylated derivative and then excreted in the urine (Liu et al., 2017b).

A recent study showed that gene polymorphisms in the *CYP3A4* and *CYP3A5* genes influence the biological availability of ticagrelor. The *CYP3A4* intron six single-nucleotide polymorphism (SNP) (rs35599367C>T, CYP3A4*22), which has an allele frequency of 3%–8% in the Caucasian population and less than 1% in the African and Asian population, reduces the hepatic expression of CYP3A4, explaining ∼12% of CYP3A4 enzyme activity variability (Wang et al., 2011; Mulder et al., 2021). Previous research has shown that CYP3A4 (CYP3A4*22) genotype correlate with the pharmacodynamics (PD) and pharmacokinetics (PK) of ticagrelor, resulting in more platelet inhibition 24 h after ticagrelor administration, consistent with a decreased metabolism and thus higher plasma concentrations (Holmberg et al., 2019). As a consequence, being carrier of the CYP3A4*22 allele could lead to an increased risk of ticagrelor-related side-effects, such as bleedings and dyspnea.

To date, the effects of *CYP3A4* and *CYP3A5* genetic polymorphisms have only been studied in trials with a small sample size and with regards to PD and PK. Little is known about the clinical effects of *CYP3A4* and *CYP3A5* polymorphisms with respect to ticagrelor efficacy. Our study aims to assess the effects of the CYP3A4*22 allele and *CYP3A5* expressor status in ticagrelor treated patients with a myocardial infarction, with respect to clinical endpoints and the most common side-effect dyspnea.

## Methods

### Study design and patient population

The rationale and design of the POPular Genetics trial have been described previously (Bergmeijer et al., 2014). In brief, the POPular Genetics was a randomized, open-label, multicenter controlled trial involving 2,488 patients with ST-segment elevation myocardial infarction (STEMI) undergoing primary percutaneous coronary intervention (PCI). Patients were randomized to *CYP2C19* genotyping or routine ticagrelor or prasugrel treatment. In the genotyping group, patients carrying a CYP2C19*2 or *3 loss-of-function allele were prescribed ticagrelor or prasugrel, and patients without a CYP2C19*2 or *3 allele received clopidogrel. Patients were followed until 1 year after admission and all endpoints were adjudicated by a blinded event committee. The aim of the study was to compare *CYP2C19* genotype-guided antiplatelet therapy to a non-tailored strategy in terms of net clinical benefit, safety and cost-effectiveness (Claassens et al., 2019). An additional blood sample was collected and stored for further (genetic) analysis. Written informed consent was obtained from each patient. The institutional review boards of all participating centers approved the protocol of the POPular Genetics study. The current study complies with the principles of the Declaration of Helsinki.

### DNA sampling

Blood samples were collected during the POPular Genetics trial from the majority of patients in both treatment groups. After completion of the trial, CYP3A4*22 (rs35599367) and CYP3A5*3 (rs776746) genotyping was performed by LGC Biosearch Technologies (Hoddesdon, United Kingdom) using a kompetitive allele specific (KASP) genotyping assay. CYP2C19*2 and *3 genotyping was already performed during the initial study (KASP thermal cycling, 2022).

### Analyses

Each patient was classified into CYP3A4*22 carrier (carrying at least one CYP3A4*22 allele) and CYP3A4*22 non-carrier, and CYP3A5 non-expressor (homozygous for the CYP3A5*3 allele) *versus* CYP3A5 expressor (CYP3A5*1/*1 or *1/*3). Patients without blood sample or incomplete genotyping results were excluded from the analyses. In addition, patients treated with clopidogrel or prasugrel were excluded.

Three different analyses were performed. Because of the previous studies showing a significant effect on platelet inhibition in CYP3A4*22 carriers we first compared CYP3A4*22 carriers with CYP3A4*22 non-carriers, irrespective of *CYP3A5* or *CYP2C19* status. In order to gain knowledge regarding the sole function of CYP3A5 the second analysis was performed in patients not carrying a CYP3A4*22 allele (CYP3A4*1/*1)*,* comparing CYP3A5 non-expressors with CYP3A5 expressors. Third, we compared fast CYP3A metabolizers (defined as patients being both CYP3A4*22 non-carrier and CYP3A5 expressor) with CYP3A reduced metabolizers (CYP3A4*22 carriers and CYP3A5 non-expressors).

### Clinical endpoints

The number of patients available for analysis was not prospectively powered and was based on the number of patients in the original trial of whom the *CYP3A4* and *CYP3A5* genotyping results were available. There were two primary endpoints: a combined thrombotic endpoint, consisting of cardiovascular death, myocardial infarction, definite stent thrombosis and stroke, and a bleeding endpoint, consisting of Platelet Inhibition and Patient Outcomes (PLATO) major and minor bleeding. Furthermore, the individual components of the thrombotic and bleeding endpoint were analyzed as secondary endpoints.

The secondary endpoint was the cessation or switching of ticagrelor to a different P2Y_12_ inhibitor due to dyspnea.

### Statistical methods

Continuous variables are presented as mean and standard deviation (SD), or median and interquartile range (IQR), based on distribution pattern. Discrete variables are presented as frequencies and percentages (%). The Mann-Whitney or student’s t-test and Chi-square test were used to compare continuous and categorical variables, respectively. A *p*-value below 0.05 was considered statistically significant. Kaplan Meier curves were estimated and used to graphically assess the primary endpoints and the log-rank test was used to calculated the *p*-values. Cox proportional hazard models were used to calculate crude and adjusted hazard ratio’s (HR) and the 95% confidence interval (CI). Logistic regression analyses were used to calculate crude and adjusted odds ratio’s (OR) and the 95% CI. To adjust for baseline differences, all baseline differences with a *p*-value <0.15 were candidate for univariateregression. If there was a significant correlation (*p < 0.10*) in the univariateanalysis, these baseline characteristics were selected for the multivariate regression analysis. All variables with a remaining *p < 0.10* in the multivariate regression analysis were considered as confounders in the regression model. All analyses were performed using SPSS version 26 (SPSS Inc., Chicago, IL, United States ).

## Results

### Patient characteristics

**FIGURE 1:**
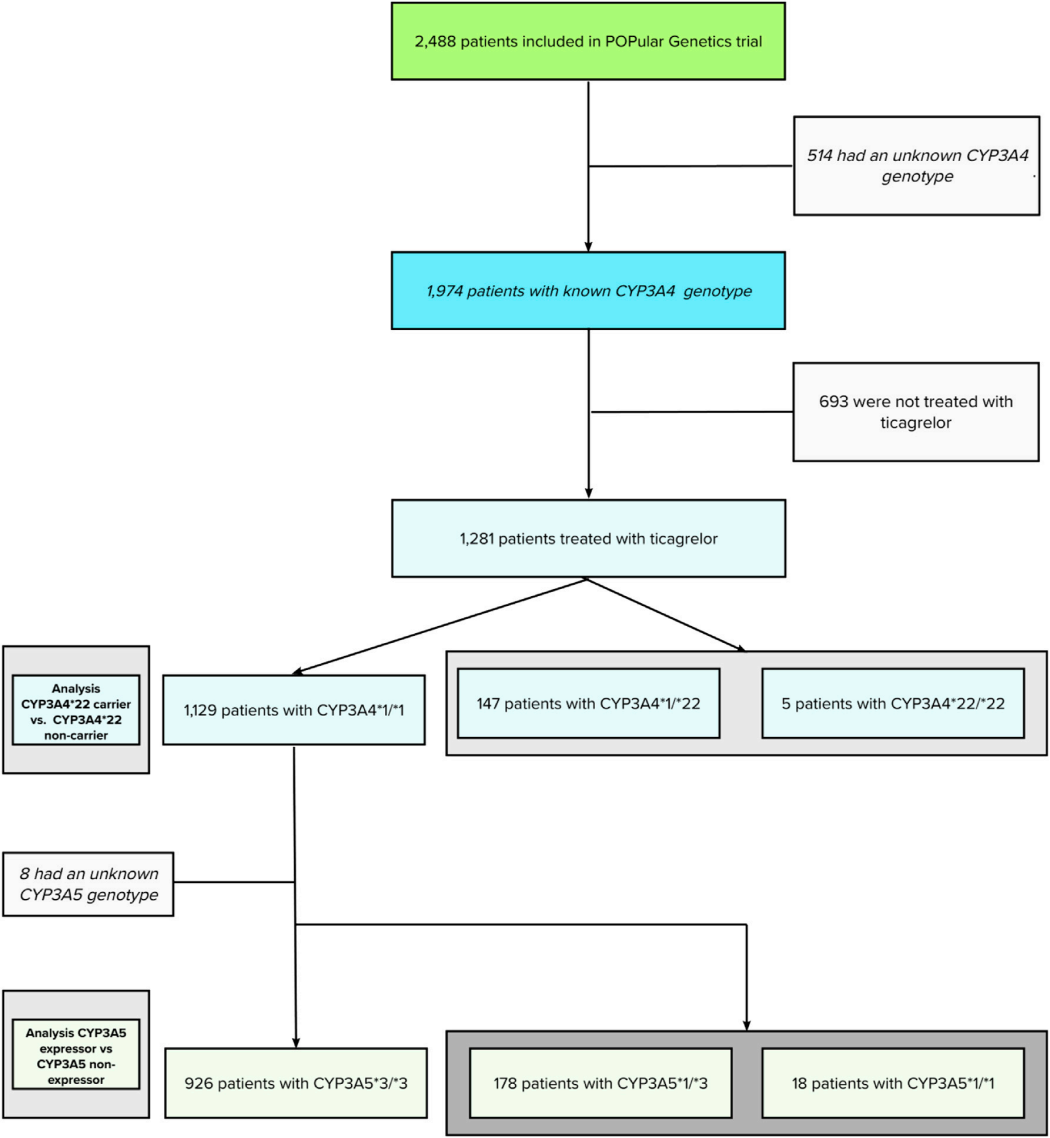
Flowchart of the POPular Genetics CYP3A4 and CYP3A5 sub study.

The initial POPular Genetics study cohort, recruiting patients from May 2012 until April 2018, consisted of 2,488 patients (100%). Using additional genotyping after study completion, CYP3A4*22 and CYP3A5*3 were successfully genotyped in 1,974 patients (79.3%). Genotypes for *CYP3A4* and *CYP3A5* could not be obtained in 514 patients (21.7%) due to assay failure or lack of a blood sample. From this cohort, a total of 1,281 patients (64.8%) were treated with ticagrelor. This patient cohort had a complete follow-up and was used for the current analyses (flowchart is presented in Figure 1).

The mean age was 61.4 ± 11.4 years; 23.4% of patients were female. A total of 147 patients (11.5%) had the CYP3A4*1/*22 genotype, five (0.4%) had the CYP3A4*22/*22 genotype. The remaining 1,129 patients (88.1%) were classified as CYP3A4*1/*1 (wild type) genotype. Furthermore, in CYP3A4*1/*1 patients, 178 patients (15.7%) had the CYP3A5*1/*3 genotype, 17 patients (1.5%) the CYP3A5*1/*1 genotype, and 926 (82.0%) patients the CYP3A5*3/*3 genotype.

In Table 1 the baseline characteristics of CYP3A4*22 carriers *versus* non-carriers are presented. In the CYP3A4*22 groups, all variables were balanced in baseline characteristics, except for a significant higher frequency of females (30.3% vs. 22.5%; *p = 0.03*), prior stroke or TIA (5.3% vs. 2.6%; *p = 0.06*), more common use of AT-II antagonists (13.2% vs. 9.0%; *p = 0.10*), and statin use (98.7% vs. 96.5%; *p = 0.15*). Furthermore, the CYP3A4*22 carriers more often had bifurcation lesions when compared to CYP3A4*22 non-carriers, (9.9% vs. 19.4%; *p = 0.01*) (Supplementary Table S1).

**TABLE 1:** Baseline characteristics of ticagrelor treated patients according to CYP3A4 status.

	POPular genetics cohort (ticagrelor treated patients)			
	All patients *N* = 1281	CYP3A4*22 non carriers^a^ *N* = 1129	*CYP3A4* *22 carriers *N* = 152	*p-value*
Age (yrs.), mean ± SD	61.4 ± 11.4	61.3 ± 11.4	62.1 ± (11.2)	*0.41*
Body mass index (kg/m2), mean ± SD	27.3 ± 6.7	27.4 ± 7.0	26.8 ± 3.8	*0.33*
Female sex, n (%)	300 (23.4)	254 (22.5)	46 (30.3)	*0.03* *
Medical history, n (%)				
Current or former smoker (%)	573 (44.7)	502 (44.4)	71 (46.7)	*0.43*
Hypertension (%)	502 (39.2)	445 (39.4)	57 (37.5)	*0.65*
Hyperlipidemia (%)	260 (20.3)	235 (20.8)	25 (16.4)	*0.21*
Diabetes mellitus (%)	139 (10.9)	120 (10.6)	19 (12.5)	*0.49*
Chronic kidney disease (%)^b^	106 (8.3)	94 (8.3)	12 (7.9)	*0.75*
Peripheral arterial disease	28 (2.2)	22 (1.9)	6 (3.9)	*0.11*
Coronary artery disease^c^	131 (10.2)	119 (10.5)	12 (7.9)	*0.31*
Relevant bleeding	29 (2.3)	26 (2.3)	3 (2.0)	*0.80*
Prior stroke or TIA	37 (2.9)	29 (2.6)	8 (5.3)	*0.06* *
Prior myocardial infarction	99 (7.7)	91 (8.1)	8 (5.3)	*0.23*
Prior PCI	99 (7.7)	90 (8.0)	9 (5.9)	*0.37*
Prior CABG	15 (1.2)	14 (1.2)	1 (0.7)	*0.53*
Clinical presentation				
Heart rate (bpm), mean ± SD	73.4 ± 14.6	73.2 ± 14.6	74.8 ± 13.2	*0.20*
Systolic BP (mmHg), mean ± SD	132.5 ± 20.4	132.6 ± 20.5	132.2 ± 19.9	*0.82*
Serum creatinine (µmol/L), mean ± SD	79.7 ± 20.4	79.9 ± 20.6	78.1 ± 18.5	*0.29*
Killip class, n (%)II-IV	9 (0.7)	8 (0.7)	1 (0.7)	*0.98*
Length of hospital stay (days), mean ± SD	3.2 ± 2.4	3.2 ± 2.5	3.1 ± 1.9	*0.73*
Discharge medication, n (%)				
Aspirin	1255 (98.0)	1104 (97.8)	151 (99.3)	*0.20*
Ticagrelor	1281 (100.0)	1129 (100.0)	152 (100.0)	*—*
Vitamin K antagonist	0 (0.0)	0 (0.0)	0 (0.0)	*—*
Novel oral anticoagulant	3 (0.2)	3 (0.3)	0 (0.0)	*0.53*
ACE inhibitor	1006 (78.5)	888 (78.7)	118 (77.6)	*0.77*
AT-II antagonist	122 (9.5)	102 (9.0)	20 (13.2)	*0.10* *
Beta blocker	1134 (88.5)	996 (88.2)	138 (90.8)	*0.35*
Statin	1239 (96.7)	1089 (96.5)	150 (98.7)	*0.15*
Proton Pump Inhibitor	958 (74.8)	840 (74.4)	118 (77.6)	*0.39*
CYP2C19 LoF carrier^d^	321 (68.6)	294 (68.9)	27 (65.9)	0.69
CYP3A4 genotype (n, %)				
*1/*1	1129 (88.1)	1129 (100.0)	0 (0.0)	*—*
*1/*22	147 (11.5)	0 (0.0)	147 (96.7)	*—*
*22/*22	5 (0.4)	0 (0.0)	5 (3.3)	*—*
CYP3A5 genotype (n,%)				
*3/*3	1065 (83.1)	926 (82.0)	139 (91.4)	*—*
*1/*3	189 (14.8)	178 (15.8)	11 (7.2)	*—*
*1/*1	18 (1.4)	17 (1.5)	1 (0.7)	*—*

TIA, transient ischemic attack; PCI, percutaneous coronary intervention; CABG, coronary artery bypass grafting; bpm, beats per minute; BP, blood pressure; ACE, indicates angiotensin-converting enzyme; AT II, angiotensin II; BMI, body mass index; creatinine clearance was calculated with the use of the CKD-EPI, formula.

^a^ CYP3A4*22 non carriers exists out of patients with the CYP3A4*1/*1 genotype, CYP3A4*22 carriers exists out of patients with the CYP3A4*1/*22 and CYP3A4*22/*22 genotype.

^b^ Chronic kidney disease are patients with an estimated glomerular filtration rate <60 ml/min/1.73 m2

^c^ Coronary artery disease is defined as an obstruction of >50% in the epicardial coronary arteries.

^d^ CYP2C19 LOF, carrier indicates the presence of CYP2C19*2 or CYP2C19*3 alleles, this was known in 427 CYP3A4*22 non-carriers and 41 carriers.

* Variables with a *p*-value <0.10 which are ued for the multivariate analysis.

Supplementary Table S2 and Supplementary Table S3 present the variables used in the univariate and multivariate regression analysis based on the *CYP3A4* status with regards to the bleeding endpoint.

In the *CYP3A5* groups, all variables were balanced in baseline characteristics, except for a significant higher prevalence of prior CABG (3.1% vs. 0.9%; *p = 0.01*).

CYP3A5 expressors had a numerically higher BMI (28.1 vs. 27.1; *p = 0.10*) and a numerically higher prevalence of prior stroke or TIA (4.1% vs. 2.2%; *p = 0.10*).

The baseline characteristics of the CYP3A5 expressor *versus* non-expressor patients can be found in Supplementary Table S1A.

Supplementary Table S4 and Supplementary Table S5 present the variables used in the univariate and multivariate regression analysis based on the CYP3A5 status with regards to the bleeding endpoint.

### Clinical impact of CYP3A4*22 carrier status

**FIGURE 2:**
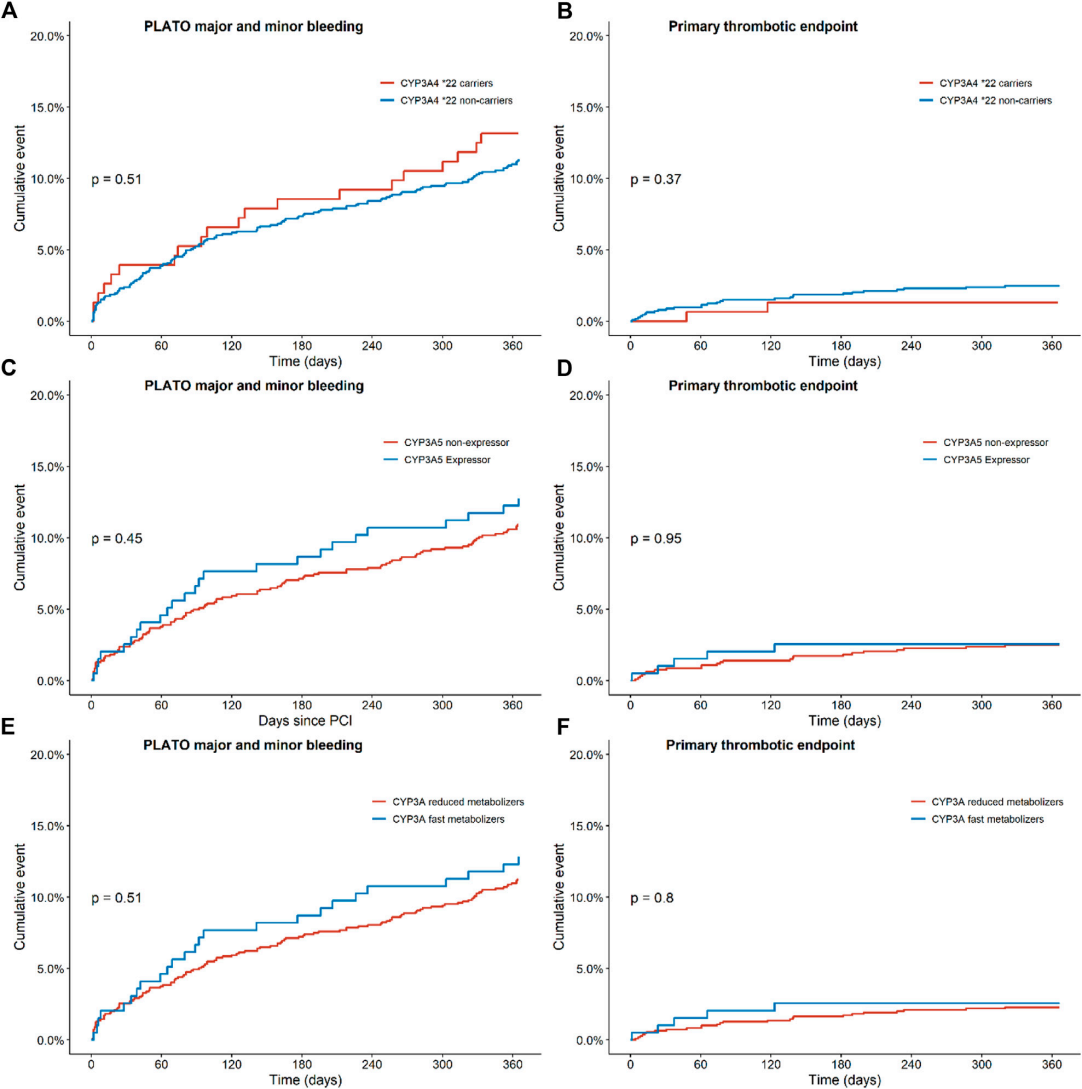
Endpoints of ticagrelor treated patients with regards to the combined thrombotic and bleeding endpoint for CYP3A, CYP3A4 and CYP3A5 status. Kaplan-Meier curves for **(A)** the combined bleeding endpoint, defined as PLATO major and minor bleeding in CYP3A4*22 carriers *versus* non-carriers, for **(B)** the combined thrombotic endpoint, defined as cardiovascular death, myocardial infarction, definite stent thrombosis, and stroke in CYP3A4*; 22 carriers *versus* non carriers, for **(C)** the combined bleeding endpoint in CYP3A5 expressors *versus* non-expressors, for **(D)** the combined thrombotic endpoint in CYP3A5 expressors *versus* non-expressors, for **(E)** the combined bleeding endpoint in CYP3A fast metabolizers *versus* reduced metabolizers and for **(F)** the combined thrombotic endpoint in CYP3A fast metabolizers *versus* reduced metabolizers.

For this analysis, 152 patients carrying a CYP3A4*22 allele and 1,129 patients with a CYP3A4*1/*1 genotype were compared (Table 2). No significant differences were observed between the two groups for the combined thrombotic endpoint [1.3% vs. 2.5%, adjusted HR 1.81 (0.43–7.62), *p* = 0.42; Figure 2B], or the combined bleeding endpoint [13.2% vs. 11.3%, adjusted HR 0.93 (0.58–1.50), *p* = 0.77; Figure 2A]. With regards to dyspnea, 194 patients switched from ticagrelor to another P2Y_12_ inhibitor or discontinued P2Y_12_-therapy. We observed no significant differences between the groups with respect to the occurrence of dyspnea: 44.0% in CYP3A4*22 carriers, and 45.0% in CYP3A4*22 non-carriers [OR 1.04 (0.45–2.42), *p* = 0.93, Supplementary Figure S6 and Supplementary Figure S7]. No multivariate analysis was performed, because no significant confounders were presented by the univariate analysis.

**TABLE 2:** Clinical endpoint for CYP3A4 in patients treated with ticagrelor.

	CYP3A4*22 carriers (*N* = 152)	CYP3A4*22 non-carriers (*N* = 1129)	Unadjusted hazard ratio (95%CI)	Adjusted hazard ratio (95%CI)¥	Adjusted *p*-value
—	***Cumulative incidence***	***Cumulative incidence***	—	—	—
Thrombotic endpoint No. Of patients (%)					
Cardiovascular death, MI, definite ST, and stroke	2 (1.3)	28 (2.5)	1.87 [0.45–7.8]	1.81 [0.43–7.62]	0.42
Cardiovascular death	0 (0.0)	6 (0.5)	—	—	0.99
Stroke	2 (1.3)	8 (0.7)	—	—	0.36
Myocardial infarction			—	—	
Spontaneous MI	2 (1.3)	41 (3.6)	—	—	0.17
Definite ST	2 (1.3)	7 (0.6)	—	—	0.99
Bleeding endpoint					
PLATO major and minor bleeding	20 (13.2)	128 (11.3)	0.85 [0.53–1.36]	0.93 [0.58–1.50]	0.77
PLATO major	4 (2.6)	16 (1.4)	—	—	0.89
PLATO minor	16 (10.5)	112 (9.9)	—	—	0.91
—	Cumulative incidence	Cumulative incidence	Unadjusted Odds Ratio	Unadjusted *p*-value	
Side effect - No. Of patients (%)					
Dyspnea^a^	11 (44.0)	76 (45.0)	1.04 [0.45–2.42]	*0.93*	—

ST, stent thrombosis; MI, myocardial infarction; PLATO, Platelet Inhibition and Patient Outcomes.

¥ Significant variables are shown in Supplementary Table S2 and Supplementary Table S3, adjustments were made for the covariate statins as confounder for the thrombotic endpoints. With regards to the bleeding endpoint adjustments were made for prior stroke and peripheral arterial disease.

a The total percentages were calculated based on all 194 patients who switched from ticagrelor to another P2Y12-inhibitor or discontinued treatment. No multivariate analysis was performed, because no significant confounders were presented by the univariate analysis.

### Clinical effect of CYP3A5 expressor

In this analysis, 196 CYP3A5 expressors (with a CYP3A5*1/1 or CYP3A5*1/*3 genotype) and 926 CYP3A5 non-expressors (patients with a CYP3A5*3/*3 genotype) were compared (Table 3). No significant differences were found between the two groups for the combined thrombotic endpoint [2.6% vs. 2.5%, adjusted HR 1.03 (0.39–2.71), *p* = 0.95; Figure 2D], or the combined bleeding endpoint [12.8% vs. 10.9%, adjusted HR 1.13 (0.73–1.76), *p* = 0.58; Figure 2C]. With respect to dyspnea, 168 patients switched from ticagrelor to another P2Y_12_ inhibitor or discontinued P2Y_12_-therapy. No significant differences were found between CYP3A5 non-expressors *versus* expressors [47.8% *versus* 35.3% OR 0.60 (0.27–1.30), *p* = 0.20; Supplementary Figure S8 and Supplementary Figure S9].

**TABLE 3:** Clinical Endpoint for CYP3A5 in patients treated with ticagrelor.

Ticagrelor treated patients with CYP3A4*22 non-carriers					
Endpoint	CYP3A5 non-expressors^a^ *N* = 926	CYP3A5 expressors^a^ *N* = 196	Unadjusted hazard ratio (95% CI) ¥	Unadjusted *p*-value	
—	Cumulative incidence	Cumulative incidence	—	—	—
Thrombotic endpoints—No. Of patients (%)					
Cardiovascular death, MI, definite ST, and stroke	23 (2.5)	5 (2.6)	1.03 [0.39–2.71]	0.95	—
Cardiovascular death	6 (0.6)	0 (0.0)	—	0.48	—
Stroke	7 (0.8)	0 (0.0)	—	0.44	—
Myocardial infarction					
Spontaneous MI	30 (3.2)	10 (5.1)	—	0.20	—
Definite ST	6 (0.6)	1 (0.5)	—	0.88	—
Bleeding endpoints	CYP3A5 non-expressors *N* = 926	CYP3A5 expressors *N* = 196	Unadjusted Hazard ratio (95% CI)	Adjusted Hazard ratio (95% CI)¥	Adjusted *p*-value
PLATO major and minor bleeding	101 (10.9)	25 (12.8)	1.15 (0.74–1.77)	1.13 (0.73–1.76)	*0.58*
PLATO major	16 (1.7)	0 (0.0)	—	—	—
PLATO minor	85 (9.2)	25 (12.8)	1.45 (0.92–2.30)	0.45 (0.92–2.29)	*0.11*
Side-effects	Cumulative incidence	Cumulative incidence	Unadjusted Odds Ratio	Unadjusted *p*-value	—
No. Of patients (%)					
Dyspnea^b^	64 (47.8)	12 (35.3)	0.60 (0.27–1.30)	0.20	—

ST, stent thrombosis; MI, myocardial infarction; PLATO, platelet inhibition and patient outcomes.

¥ No confounders were chosen based on the univariate and multivariable cox regression models for the individual trombotic endpoints. For the bleeding endpoints the covariate prior stroke or TIA was used to adjust for confounders, see Supplementary Table S4 and Supplementary Table S5.

^a^ CYP3A5 non-expressors are patients with the genotype CYP3A5*3/*3

^b^ The total percentages were calculated based on all 168 patients who switched from ticagrelor to another P2Y12-inhibitor or discontinued treatment.

### Clinical effect of CYP3A fast metabolizers

In this analysis, 195 CYP3A fast metabolizers (with both a CYP3A4*1/*1 and CYP3A5*1/1 or CYP3A5*1/*3 genotype) and 1,094 CYP3A reduced metabolizers (patients with a CYP3A4*1/*22 or CYP3A4*22/*22 and CYP3A5*3/*3 genotype) were compared (Table 4). No significant differences were found between the two groups for the combined thrombotic endpoint [2.6% vs. 2.3%, HR 1.13 (0.43–2.95), *p* = 0.81; Figure 2F], or the combined bleeding endpoint [12.8% vs. 11.2%, adjusted HR 1.13 (0.73–1.73), *p* = 0.59; Figure 2E]. With respect to dyspnea, 195 patients switched from ticagrelor to another P2Y_12_ inhibitor or discontinued P2Y_12_-therapy. No significant differences were found between CYP3A fast metabolizers *versus* reduced metabolizers [35.3% *versus* 47.2%, OR 0.60 (0.28–1.32), *p* = 0.21; Table 4].

**TABLE 4:** Clinical endpoint for CYP3A fast metabolizers *versus* CYP3A reduced metabolizers in patients treated with ticagrelor.

	*CYP3A fast metabolizers* *^a^* (*N* = 195)	*CYP3A reduced metabolizers* *^a^* (*N* = 1094)	Hazard ratio (95% CI)	*p*-value
Thrombotic endpoint No. Of patients (%)				
—	*Cumulative incidence*	*Cumulative incidence*	—	—
Cardiovascular death, MI, definite ST, and stroke	5 (2.6)	25 (2.3)	1.13 (0.43–2.95)	0.81
Cardiovascular death	0 (0.0)	6 (0.6)	—	0.17
Stroke	0 (0.0)	9 (0.8)	—	0.09
Myocardial infarction			—	
Spontaneous MI	5 (2.6)	13 (1.2)	—	0.17
Definite ST	1 (0.5)	8 (0.7)	—	0.99
Thrombotic endpoint No. Of patients (%)				
PLATO major and minor bleeding	25 (12.8)	123 (11.2)	1.13 (0.73–1.73)	0.59
PLATO major	0 (0.0)	20 (1.8)	—	0.06
PLATO minor	25 (12.8)	103 (9.4)	—	0.15
—	Cumulative incidence	Cumulative incidence	Unadjusted Odds Ratio	—
Dyspnea^b^	12 (35.3)	76 (47.2)	0.61 (0.28–1.32)	0.21

ST, stent thrombosis; MI, myocardial infarction; PLATO, platelet inhibition and patient outcomes.

^a^ CYP3A fast metabolizers is defined by CYP3A4*22 non-carriers and CYP3A5 non-expressors. CYP3A4 reduced metabolizers is defined by CYP3A4*22 carriers and CYP3A5 expressors.

^b^ The total percentages were calculated based on all 195 patients who switched from ticagrelor to another P2Y12-inhibitor or discontinued treatment.

## Discussion

In this analysis, STEMI patients treated with ticagrelor who were carrier of a CYP3A4*22 allele showed no statistical significant difference in thrombotic or bleeding rates compared to CYP3A4*22 non-carriers. The same holds true for patients who were *CYP3A5* expressor *versus CYP3A5* non-expressor, adjusted for CYP3A4*22 genotype (as only CYP3A4*22 non-carriers were considered), and for CYP3A fast metabolizer *versus* CYP3A reduced metabolizer patients. Additionally, there was no significant difference in the rate of dyspnea in relation to the SNP’s mentioned. Nevertheless, the number of patients with a thrombotic event was numerically higher in the CYP3A4*22 non-carrier group, while bleeding was numerically lower. While no definite conclusions can be drawn based on a numerical trend, our data shows a direction of effect as was expected based on the rationale of the study analysis. Although our study population was relatively large, the number of patients carrying a CYP3A4*22 allele was low, and therefore a recessive model had to be used (comparing CYP3A4*1/*1 to *1/*22 plus *22/*22), diluting a possible effect of the CYP3A4*22 polymorphism. Analysis in a much larger patient cohort, using a dominant gene model, will be necessary to achieve adequate statistical power to draw definite conclusion.

### CYP3A4*22 carriers

Earlier pharmacodynamic studies demonstrated a significant increase in ticagrelor concentration in CYP3A4*22 carriers; however, these studies did not evaluate clinical endpoints for the CYP3A4*22 allele and CYP3A5 expressor status (Kreutz et al., 2013; Varenhorst et al., 2015a). For example, a study performed by Holmberg et al. (2019) in 19 healthy volunteers with the CYP3A4*1/*1 genotype and six with the CYP3A4*1/*22 genotype found that the AUC of ticagrelor was 89% higher in CYP3A4*22 carriers than in *22 non-carriers, and *22 carriers showed more pronounced platelet inhibition (antiplatelet activity was tested with turbidimetric optical detection using the VerifyNow) 24 h after administration of a single dose of ticagrelor (43% vs. 21%, *p* = 0.029). They concluded that the CYP3A4*22 allele markedly impairs ticagrelor elimination, enhancing its antiplatelet effect, which could potentially lead to a higher risk for bleeding.

### CYP3A5 expressor status

Liu et al. (2017a) studied the effect of CYP3A5*3 on platelet reactivity. Only a minor impact of CYP3A5*3 on platelet reactivity was found, which led the authors to conclude that there should be no dosage adjustment based on this allele.

### Other genetic influences on ticagrelor

Varenhorst et al. (2015a) performed a genome-wide association study (GWAS) with patients treated with ticagrelor in the PLATO trial, which was the landmark trial demonstrating the clinical effect of ticagrelor in ACS patients (James et al., 2011). Using a discovery cohort (*n* = 1,812) and a replication cohort (*n* = 1,941), three genetic loci were found to be of genome wide significance (*CYP3A4*, *SLC O 1B1*, *UGT2B7*) for ticagrelor pharmacokinetics in this ACS patient cohort. The modest effects of these loci on ticagrelor plasma levels did not translate into any detectable effect on the primary composite endpoints, non-CABG-related bleeding, or patient-reported dyspnea. The *CYP3A4* SNP included in the GWAS, however, was the CYP3A4*7 allele (rs56324128), while the CYP3A4*22 allele was not included (Eiselt et al., 2001). The CYP3A4*22 (rs35593367C>T) polymorphism results in an amino acid substitution and has been shown to impair the enzymatic function of CYP3A4, which result in a reduction in the elimination of ticagrelor; the function of the CYP3A4*7 allele is unkown (Nieuweboer et al., 2012; Mulder et al., 2021). In this study CYP3A4*7 was not determined, and therefore was not taken into account.

There are currently no other studies known to the authors that evaluated the influence of CYP3A4*22 or CYP3A5*3 on clinical endpoints in patients using ticagrelor (Mulder et al., 2021).

### Clinical relevance

Treatment regimens based on pharmacogenetic data are used more often in clinical practise, and have been shown to be important in STEMI patients treated with clopidogrel, based on *CYP2C19* genotype (Claassens et al., 2019). While treatment with antiplatelet drugs is a constant balancing act between efficacy (preventing thrombotic events) and safety (preventing bleeding events), any new factors influencing this balance might help to find the optimal balance for the individual patient (Varenhorst et al., 2015b).

### Limitations

This study has several important limitations. First, this was a sub study of a larger randomized trial; therefore, it was not powered for the primary endpoints. As mentioned above with respect to CYP3A4*22 analysis, the low number of patients being homozygous for the CYP3A4*22 allele led to the use of a recessive model (comparing CYP3A4*1/*1 to *1/*22 and *22/*22) instead of a dominant model. Therefore, a possible effect limited to patients homozygous for the CYP3A4*22 allele or CYP3A5 expressor status might have been underestimated or missed. Second, the use of strong CYP3A4 inhibitors or inducers, other than statins, could not be accounted for, and could potentially have influenced the results. Finally, Varenhorst et al. (2015b) found an association between other genetic loci, such as *UGT2B7* and *SLCO1B1,* and plasma ticagrelor levels, although there is no direct evidence for whether *UGT2B7* or *SLCO1B1* is involved in ticagrelor metabolism. Those SNP’s were not available for analysis in our patient cohort. In addition, the POPular Genetics trial randomized patients to genotyping *versus* conventional treatment. Therefore, *CYP2C19* genotype is not normally distributed over this study group, supposing that CYP2C19 has no clinically relevant effect on ticagrelor there could still be a possible effect on *CYP3A4* or *CYP3A5*. The baseline shows that there is no significant difference between the two groups. Therefore, no further adjustments were made and the clinical effect is expected to be minimal.

Although the above limitations are clear, we feel that the unique nature of these data on SNP’s in relation to clinical endpoints in ticagrelor treated ACS patients contributes to the understanding of the genetic influences of the CYP3A locus on ticagrelor metabolism and their impact on clinical endpoints.

## Study highlights

What is the current knowledge on the topic?

No studies are known examining the clinical effect of the genetic variations of *CYP3A4* and *CYP3A5* with respect to ticagrelor efficacy.

What question did this study address?

Our study assessed the effects of the CYP3A4*22 allele and *CYP3A5* expressor status in ticagrelor treated patients with a myocardial infarction, with respect to clinical endpoints and the side-effect dyspnea.

What does this study add to our knowledge?

STEMI patients treated with ticagrelor who were carrier of a CYP3A4*22 allele showed no statistical significant difference in thrombotic or bleeding rates compared to CYP3A4*22 non-carriers. The same holds true for patients who were *CYP3A5* expressor *versus CYP3A5* non-expressor, and for CYP3A fast metabolizer *versus* CYP3A reduced metabolizer patients. No significant difference in the rate of dyspnea in relation to the SNP’s was seen.

How might this change clinical pharmacology or translational science?

The current results will not lead to changes in clinical practice. Conducting studies with a larger cohort and using a dominant gene model will be necessary to achieve adequate statistical power.

## Conclusion

The CYP3A4*22 polymorphisms and CYP3A5 expressor status in ticagrelor treated patients presenting with STEMI did not show a statistical significant association with bleeding or thrombotic events in this analysis. In addition, no association was found between *CYP3A4* or *CYP3A5* genotypes and dyspnea.

## Data Availability

The datasets presented in this study can be found in online repositories. The name of the repository is RedCap. This is not mentioned in the article nor in the Supplementary Material. Data can be requested through JT through jurtenberg@gmail.com.
