# Supplementary material for: Effects of CYP3A4*22 and CYP3A5 on clinical outcome in patients treated with ticagrelor for ST-segment elevation myocardial infarction: POPular Genetics sub-study
Source: Front Pharmacol. 2022 Dec 5;13:1032995. doi: 10.3389/fphar.2022.1032995 (PMC9760790; doi:10.3389/fphar.2022.1032995)
Supplement: Supplementary file 1 [file DataSheet1.pdf]

<u>Supplementary Appendix</u>

Effects of *CYP3A4*22* carrier status and CYP3A5 expressor status on clinical outcome in patients treated with ticagrelor for ST-segment elevation myocardial infarction

POPular Genetics CYP3A4 and CYP3A5 genotype sub-study

**Supplementary table 1**	Baseline procedural characteristics of ticagrelor treated patients according to CYP3A4 status
**Supplementary table 1a.**	Baseline CYP3A5 expressors vs. CYP3A5 non-expressors
**Supplementary table 2.**	Univariable Cox Regression Analysis for PLATO major and minor bleeding (non-CABG) based on CYP3A4 status.
**Supplementary table 3.**	Multivariate Cox Regression Analysis for PLATO major and minor bleeding (non-CABG) based on CYP3A4 status.
**Supplementary table 4.**	Univariable Cox Regression Analysis for PLATO major and minor bleeding based on CYP3A5 status.
**Supplementary table 5.**	Multivariate Cox Regression Analysis for PLATO major and minor bleeding based on CYP3A5 status.
**Supplementary table 6.**	Univariable logistic Regression Analysis for dyspnoea based on CYP3A4 baseline variabeles.
**Supplementary table 7.**	Multivariate Logistic Regression Analysis for dyspnoea based on CYP3A4 baseline variabeles.
**Supplementary table 8.**	Univariate Logistic Regression Analysis for dyspnoea based on CYP3A5 baseline variables.
**Supplementary table 9.**	Multivariate Logistic Regression Analysis for dyspnoea based on CYP3A5 baseline variables.

Table 1. Baseline characteristics of ticagrelor treated patients according to CYP3A4 status

POPular Genetics cohort (ticagrelor treated patients)

	All patients N=1281	*CYP3A4*22* non carriers[a] N=1129	*CYP3A4*22* carriers N=152	*P-value*
**Procedural characeristics, n (%)**				
**Access site**				*0.91*
**Brachial**	1 (0.1)	1 (0.1)	0 (0.0)	
**Femoral**	354 (27.6)	312 (27.6)	42 (27.6)	
**Radial**	923 (72.1)	813 (72.0)	110 (72.4)	
**Multivessel disease**	616 (48.1)	548 (48.5)	68 (44.7)	*0.38*
**Stent**				
**None**	19 (1.5)	18 (1.6)	1 (0.7)	*0.37*
**Bare metal stent**	47 (3.7)	42 (3.7)	5 (3.3)	*0.79*
**Bioresorbable scaffold**	11 (0.9)	11 (1.0)	0 (0.0)	*0.22*
**Drug eluting stent**	1220 (95.2)	1073 (95.0)	147 (96.7)	*0.36*
**Total stent length, mm, mean ± SD**	27.5 ± 23.0	27.5 ± 23.0	26.9 ± 16.8	*0.59*
**Bifurcation lesion**	234 (18.3)	219 (19.4)	15 (9.9)	*0.01**
**Ostial lesion**	75 (5.9)	68 (6.0)	7 (4.6)	*0.68*

[a]*CYP3A4**22 non carriers exists out of patients with the *CYP3A4**1/*1 genotype, *CYP3A4**22 carriers exists out of patients with the *CYP3A4**1/*22 and *CYP3A4**22/*22 genotype. *Variables with a p-value <0.10 which are used for the multivariate analysis

Supplementary table 1a. Baseline characteristics of ticagrelor treated patients according to CYP3A5 status

**POPular Genetics cohort (ticagrelor treated patients) with CYP3A4*1/*1 genotype**			***P-value***
	**CYP3A5 non-expressors N=926**	**CYP3A5 expressors N=196**	
Age (yrs.), mean ± SD	61.4 (11.6)	61.2 ± (10.2)	*0.80*
Body mass index (kg/m²), mean ± SD	27.2 ± 4.4	28.1 ± 13.8	*0.10**
Female sex, n (%)	213 (23.0)	40 (20.4)	*0.43*
**Medical history, n(%)**			
Current or former smokers (%)	414 (44.7)	86 (43.9)	*0.98*
Hypertension (%)	561 (60.6)	76 (38.8)	*0.87*
Hyperlipidemia (%)	739 (79.8)	47 (24.0)	*0.24*
Diabetes mellitus (%)	823 (88.9)	17 (8.7)	*0.31*
Chronic kidney disease (%)	76 (8.2)	16 (8.2)	*0.92*
Peripheral arterial disease	18 (1.9)	4 (2.0)	*0.93*
Coronary artery disease	93 (10.0)	26 (13.3)	*0.18*
Relevant bleeding	22 (2.4)	4 (2.0)	*0.78*
Prior stroke or TIA	20 (2.2)	8 (4.1)	*0.10**
Prior MI	73 (7.9)	18 (9.2)	*0.55*
Prior PCI	71 (7.7)	19 (9.7)	*0.34*
Prior CABG	8 (0.9)	6 (3.1)	*0.01**
**Clinical presentation**			

Heart rate (bpm) , mean ± SD	73.2 ± 14.6	74.8 ± 13.2	*0.93*
Systolic BP (mmHg) , mean ± SD	132.8 ± 20.5	131.6 ± 20.5	*0.44*
Serum creatinine (µmol/L) , mean ± SD	79.7 ± 20.9	80.9 ± 19.0	*0.46*
ST-segment deviation, n (%)			
ST-elevation	908 (98.1)	195 (99.5)	*0.36*
ST-depression	760 (82.1)	163 (83.2)	*0.42*
Killip class, n (%)			*0.45*
I	914 (98.7)	194 (99.0)	
II-IV	6 (0.6)	2 (1.0)	
Clinical diagnosis, n (%)			-
Unstable angina	0 (0.0)	0 (0.0)	
NSTEMI	0 (0.0)	0 (0.0)	
STEMI	926 (100.0)	152 (100.0)	
Length of hospital stay (days), mean ± SD	3.2 ± 2.6	3.0 ± 1.9	*0.42*
**Procedural characeristics, n (%)**			
Access site			*0.14*
Brachial	0 (0.0)	1 (0.5)	
Femoral	260 (28.1)	52 (26.5)	
Radial	663 (71.6)	143 (73.0)	
Multivessel disease	444 (47.9)	100 (51.0)	*0.43*
Stent			
None	13 (1.4)	5 (2.6)	*0.25*
Bare metal stent	34 (3.7)	8 (4.1)	*0.78*
Biovascular scaffold	9 (1.0)	2 (1.0)	*0.95*

Drug eluting stent	879 (94.9)	187 (95.4)	*0.78*
Total stent lenght, mm, mean ± SD	27.5 ± 23.0	27.0 ± 15.1	*0.61*
Bifurcation lesion	169 (18.3)	48 (24.5)	*0.06**
Ostial lesion	55 (5.9)	13 (6.6)	*0.45*
**Discharge medication, n (%)**			
Aspirin	904 (97.6)	193 (98.5)	*0.47*
Ticagrelor	926 (100.0)	196 (100.0)	-
Vitamin K antagonist	0 (0.0)	0 (0.0)	-
Novel oral anticoagulant	3 (0.3)	0 (0.0)	*0.43*
ACE inhibitor	727 (78.5)	156 (79.6)	*0.74*
AT-II antagonist	86 (9.3)	15 (7.7)	*0.47*
Beta blocker	818 (88.3)	172 (87.8)	*0.82*
Statin	894 (96.5)	189 (96.4)	*0.94*
Proton Pump Inhibitor	693 (74.8)	142 (72.4)	*0.49*
Calcium antagonist	93 (10.0)	25 (12.8)	*0.26*
CYP2C19 LoF carrier*	61 (76.3)	233 (53.3)	0.11
**CYP3A4 genotype (n, %)**			
*1/*1	926 (100.0)	196 (100.0)	-
*1/*22	-	-	-
*22/*22	-	-	-
**CYP3A5 genotype (n,%)**			
*3/*3	926 (100.0)	-	-
*1/*3	-	178 (90.8)	-
*1/*1	-	18 (9.2)	-

ACE indicates angiotensin-converting enzyme; AT II, angiotensin II; BMI, body mass index; creatinine clearance was calculated with the use of the CKD-EPI formula.

* CYP2C19 LOF carrier indicates the presence of *CYP2C19*2* or *CYP2C19*3* alleles, this was known in 427 *CYP3A4*22* non-carriers and 41 carriers

Supplementary table 2. Univariable Cox Regression Analysis for PLATO major and minor bleeding (non-CABG) based on CYP3A4 status. In bold are the variables with a p-value ≤ 0.10.

**Variable**	***p-value***	**Exp(β)**	**95% CI lower**	**95% CI upper**
Gender	*1.00*	1.00	0.69	1.45
**Peripheral arterial disease**	***0.10***	**0.51**	**0.23**	**1.17**
**Prior stroke or TIA**	***0.10***	**0.55**	**0.27**	**1.13**
AT-II antagonist	*0.51*	1.22	0.68	2.21
Statin	*0.56*	1.28	0.56	2.89

Supplementary table 3. Multivariate Cox Regression Analysis for PLATO major and minor bleeding (non-CABG) based on CYP3A4 status. In bold are the variables with a p-value ≤ 0.10.

**Variable**	***p-value***	**Exp(β)**	**95% CI lower**	**95% CI upper**
Gender	*0.96*	1.01	0.70	1.46
**Peripheral arterial disease**	***0.10***	**0.55**	**0.24**	**1.27**
**Prior stroke**	***0.09***	**0.57**	**0.27**	**1.19**
AT-II antagonist	*0.31*	1.37	0.75	2.50
Statin	*0.49*	1.33	0.59	3.03

Supplementary table 4. Univariable Cox Regression Analysis for PLATO major and minor bleeding based on CYP3A5 status. In bold are the variables with a p-value < 0.10

**Variable**	***p-value***	**Exp(β)**	**95% CI lower**	**95% CI upper**
BMI	*1.00*	0.99	0.94	1.05
**Prior stroke or TIA**	***0.10***	**0.55**	**0.27**	**1.13**
Prior CABG	*0.65*	1.59	0.22	11.36
Bifurcation lesion	*0.68*	0.92	0.62	1.37

Supplementary table 5. Multivariate Logistic Regression Analysis for PLATO major and minor bleeding based on CYP3A5 status. In bold are the variables with a p-value < 0.10

**Variable**	***p-value***	**Exp(β)**	**95% CI lower**	**95% CI upper**
BMI	*0.13*	0.97	0.93	1.01
**Prior stroke or TIA**	***0.07***	**0.51**	**0.25**	**1.05**
Prior CABG	*0.61*	1.67	0.23	11.94
Bifurcation lesion	*0.83*	0.96	0.64	1.44

Supplementary table 6. Univariable Cox Regression Analysis for dyspnea based on CYP3A4 baseline variabeles.

**Variable**	***p-value***	**Exp(β)**	**95% CI lower**	**95% CI upper**
Gender	*0.96*	0.98	0.52	1.87
Peripheral arterial disease	*0.82*	0.83	0.16	4.20
Prior stroke or TIA	*0.82*	0.83	0.16	4.20
AT-II antagonist	*0.26*	0.59	0.24	1.47

Supplementary table 7. Multivariate Logistic Regression Analysis for dyspnea based on CYP3A4 baseline variabeles.

**Variable**	***p-value***	**Exp(β)**	**95% CI lower**	**95% CI upper**
Gender	*0.79*	0.92	0.47	1.77
Peripheral arterial disease	*0.88*	0.88	0.17	4.60
Prior stroke or TIA	*0.85*	0.85	0.16	4.47
AT-II antagonist	*0.60*	0.58	0.23	1.47

Supplementary table 8. Univariable Logistic Regression Analysis for dyspnea based on CYP3A5 baseline variables.

**Variable**	***p-value***	**Exp(β)**	**95% CI lower**	**95% CI upper**
BMI	*0.21*	104	0.98	1.11
Prior stroke or TIA	*0.82*	0.83	0.16	4.20
Prior CABG	*0.26*	0.27	0.03	2.65
Bifurcation lesion	*0.83*	1.08	0.54	2.16

Supplementary table 9. Multivariate Logistic Regression Analysis for dyspnea based on CYP3A5 baseline variables.

**Variable**	***p-value***	**Exp(β)**	**95% CI lower**	**95% CI upper**
BMI	*0.26*	1.04	0.97	1.10
Prior stroke or TIA	*0.82*	0.83	0.16	4.23
Prior CABG	*0.31*	0.30	0.03	3.02
Bifurcation lesion	*1.00*	1.00	0.50	2.02
